# A Grammar-Based Semantic Similarity Algorithm for Natural Language Sentences

**DOI:** 10.1155/2014/437162

**Published:** 2014-04-10

**Authors:** Ming Che Lee, Jia Wei Chang, Tung Cheng Hsieh

**Affiliations:** ^1^Department of Computer and Communication Engineering, Ming Chuan University, Taoyuan 333, Taiwan; ^2^Department of Engineering Science, National Cheng Kung University, Tainan 701, Taiwan; ^3^Department of Visual Communication Design, Hsuan Chuang University, Hsinchu 300, Taiwan

## Abstract

This paper presents a grammar and semantic corpus based similarity algorithm for natural language sentences. Natural language, in opposition to “artificial language”, such as computer programming languages, is the language used by the general public for daily communication. Traditional information retrieval approaches, such as vector models, LSA, HAL, or even the ontology-based approaches that extend to include concept similarity comparison instead of cooccurrence terms/words, may not always determine the perfect matching while there is no obvious relation or concept overlap between two natural language sentences. This paper proposes a sentence similarity algorithm that takes advantage of corpus-based ontology and grammatical rules to overcome the addressed problems. Experiments on two famous benchmarks demonstrate that the proposed algorithm has a significant performance improvement in sentences/short-texts with arbitrary syntax and structure.

## 1. Introduction

Natural language, a term in opposition to artificial language, is the language used by the general public for daily communication. An artificial language is often characterized by self-created vocabularies, strict grammar, and a limited ideographic range and therefore belongs to a linguistic category that is less easy to be accustomed to, yet not difficult to be mastered by the general public. A natural language is inseparable from the entire social culture and varies constantly over time; individuals can easily develop a sense of this first language while growing up. In addition, the syntactic and semantic flexibility of a natural language enables this type of language to be natural to human beings. However, due to its endless exceptions, changes, and indications, a natural language also becomes the type of language that is the most difficult to be mastered.

Natural language processing (NLP) studies how to enable a computer to process and understand the language used by human beings in their daily lives, to comprehend human knowledge, and to communicate with human beings in a natural language. Applications of NLP include information retrieval (IR), knowledge extraction, question-answering (QA) systems, text categorization, machine translation, writing assistance, voice identification, composition, and so on. The development of the Internet and the large production of digital documents have resulted in an urgent need for intelligent text processing, and the theory as well as the skill of NLP has therefore become more important.

Traditionally, techniques for detecting similarity between texts have centered on developing document models. In recent years, several types of document models have been established, such as the Boolean model, the vector-based model, and the statistical probability model. The Boolean model achieves the coverage of keywords using the intersection and union of sets. The Boolean algorithm is prone to be misused and thus, a retrieval method that approximates a natural language is a direction for further improvement. Salton and Lesk first proposed the retrieval system of a vector space model (VSM) [1–3], which was not only a binary comparison method. The primary contribution of this method was in suggesting the concepts of partial comparison and similarity, so that the system can calculate the similarity between a document and a query based on the different weights of index terms, and further output the result of retrieval ranking. Concerning the actualization of a vector model, first users' queries and documents in a database should be transformed into vectors in the same dimension. While both the documents and queries are represented by the same vector space dimension, the most common evaluation on semantic similarity in a high dimensional space is to calculate the similarity between two vectors using cosine, whose value should fall between 0 and 1. Overall, the advantages of a vector space model include the following. (1) With given weights, VSM can better select characteristics, and the retrieval efficacy is largely improved compared to the Boolean model. (2) VSM provides the mechanism of partial comparison, which enables the retrieval of documents with the most similar distribution. Wu et al. present a VSM-based FAQ retrieval system. The vector elements are composited by the question category segment and the keyword segment [4]. A phrase-based document similarity measure is proposed by Chim and Deng [5]. In [5], the TF-IDF weighted phases in Suffix Tree [6, 7] are mapped into a high dimensional term space of the VSM. Very recently, Li et al. [8] presented a novel sentence similarity computation measure. Their measure, taking the semantic information and word order into account, which acquired good performance in measuring, is basically a VSM-based model.

A need for a method of semantic analysis on shorter documents or sentences has gradually occurred in the fields of NLP applications in recent years [9]. With regard to the applications in text mining, the technique of semantic analysis of short texts/sentences can also be applied in databases as a certain assessment standard to look for undiscovered knowledge [10]. Furthermore, the technique of semantic analysis of short texts/sentences can be employed in other fields, such as text summarization [11], text categorization [12], and machine translation [13]. Recently, a concept under development emphasizes that the similarity between texts is the “latent semantic analysis (LSA), which is based on the statistical data of vocabulary in a large corpus. LSA and the hyperspace analog to language (HAL) are both famous corpus-based algorithms [14–16]. LSA, also known as latent semantic indexing (LSI), is a fully automatic mathematical/statistical technique that analyzes a large corpus of natural language text and a similarity representation of words and text passages. In LSA, a group of terms representing an article was extracted by judging from among many contexts, and a term-document matrix was built to describe the frequency of occurrence of terms in documents. Let *M* be a term-document matrix where element (*x*, *y*) normally describes the* TF-IDF* weight of term *x* in document*y*. Then, the matrix representing the article is divided by singular value decomposition (SVD) into three matrices, including a diagonal matrix of SVD [15]. Through the SVD procedure, smaller singular values can be eliminated, and the dimension of the diagonal matrix can also be reduced. The dimension of the terms included in the original matrix can be decreased through the reconstruction of SVD. Through the processes of decomposition and reconstruction, LSA is capable of acquiring the knowledge of terms expressed by the article. When the LSA is applied to calculating the similarity between texts, the vector of each text is transformed into a reduced dimensional space, while the similarity between two texts is obtained from calculating the two vectors of the reduced dimension [14]. The difference between vector-based model and LSA lies in that LSA transforms terms and documents into a latent semantic space and eliminates some noise in the original vector space.

One of the standard probabilistic models of LSA is the probabilistic latent semantic analysis (PLSA), which is also known as probabilistic latent semantic indexing (PLSI) [17]. PLSA uses mixture decomposition to model the cooccurrence words and documents, where the probabilities are obtained by a convex combination of the aspects. LSA and PLSA have been widely applied in information processing systems and other applications [18–24].

The other important study based on a corpus is the hyperspace analog to language (HAL) [25]. HAL and LSA share very similar attributes: they both use concurrent vocabularies to retrieve the meaning of a term. In contrast to LSA, HAL uses a paragraph or document as a unit of the document to establish the information matrix of a term. HAL establishes a window matrix of a shared term as a basis and shifts the window width without exceeding the original definition of the window matrix. The window scans through an entire corpus, using *N* terms as the width of the term window (normally a width of 10 terms), and further forms a matrix of *N* × *N*. When the window shifts and scans the documents in the entire corpus, elements in the matrix may record the weight of each shared term (number of occurrence/frequency). A 2*N* dimensional vector of a term can be acquired by combining the lines and rows of the matrix corresponding to the term, and the similarity between two texts can be calculated by the approximate Euclidean distance. However, HAL has less satisfactory results than LSA when calculating short texts.

To conclude, the aforementioned approaches calculate the similarity based on the number of shared terms in articles, instead of overlook the syntactic structure of sentences. If one applies the conventional methods to calculate the similarity between short texts/sentences directly, some disadvantages may arise.

1. The conventional methods assume that a document has hundreds or thousands of dimensions, transferring the short texts/sentences into a very high dimensional space and extremely sparse vectors may lead to a less accurate calculation result.
2. Algorithms based on shared terms are suitable to be applied to the retrieval of medium and longer texts that contain more information. In contrast, information of shared terms in short texts or sentences is rare and even inaccessible. This may cause the system to generate a very low score on semantic similarity, and this result cannot be adjusted by a general smoothing function.
3. Stopwords are usually not taken into consideration in the indexing of normal IR systems. Stopwords do not have much meaning when calculating the similarity between longer texts. However, they are unavoidable parts with regard to the similarity between sentences, for that they deliver information concerning the structure of sentences, which has a certain degree of impact on explaining the meanings of sentences.
4. Similar sentences may be composed of synonyms; abundant shared terms are not necessary. Current studies evaluate similarity according to the cooccurring terms in the texts and ignore syntactic information.

The proposed semantic similarity algorithm addresses the limitations of these existing approaches by using grammatical rules and the WordNet ontology. A set of grammar matrices is built for representing the relationships between pairs of sentences. The size of the set is limited to the maximum number of selected grammar links. The latent semantic of words is calculated via a WordNet similarity measure. The rest of this paper is organized as follows.  Section 2 introduces related technologies adopted in our algorithm.  Section 3 outlines the proposed algorithm and core functions.  Section 4 gives some examples to illustrate our method. Experimental results on two famous benchmarks are shown in Section 5, and the final gives the conclusion.

## 2. Background

### 2.1. Ontology and the WordNet

The issue of semantic aware among texts/natural-languages is increasingly pointing towards Semantic Web technologies in general and ontology in particular as a solution. Ontology is a philosophical theory about the nature of being. Artificial intelligence researchers, especially the knowledge acquisition and representation, reincarnate the term to express “*a shared and common understanding of some domain that can be communicated between people and application systems*” [26, 27]. A typical ontology is a taxonomy defining the classes in a specific domain and their relationships as well as a set of inference rules powering its reasoning functions [28]. Ontology is now recognized in the semantic web community as a term that refers to the shared understanding of knowledge in some domains of interest [29–31], which is often conceived as a set of concepts, relations, functions, axioms, and instances. Guarino conducted a comprehensive survey for the definition of ontology from various highly cited works in the knowledge sharing community [32–37]. The semantic web is an evolving extension of the World Wide Web in which web content can be expressed in natural languages and in a form that can be understood, interpreted, and used by software agents. Elements of the semantic web are expressed in formal specifications, which include the resource description framework [38], a variety of data interchange formats (such as RDF/XML, N3, Turtle, and N-Triples) [39, 40], and notations such as web ontology language [41] and the RDF schema.

In recent years, the WordNet [42] has become the most widely used lexical ontology of English. The WordNet was developed and has been maintained by the Cognitive Science Laboratory at Princeton University in the 1990s. Nouns, verbs, adjectives, and adverbs are grouped into cognitive synonyms called “synsets,” and each synonym expresses a distinct concept. As an ordinary online dictionary, WordNet lists subjects along with explanation alphabetically. Additionally, it also shows semantic relations among words and concepts. The latest version of WordNet is 3.0, which contains more than 150,000 words and 110,000 synsets. In WordNet, the lexicalized synsets of nouns and verbs are organized hierarchically by means of hypernym/hypernymy and hyponym/hyponymy. Hyponyms are concepts that describe things more specifically, and hypernyms refer to concepts that describe things more general. In other words, *x* is a hypernym of *y* if every *y* is a kind of *x*, and *y* is a hyponym of *x* if every *y* is a kind of *x*. For example,* bird* is a hyponymy of* vertebrate*, and* vertebrate* is a hypernym of* bird*. The concept hierarchy of WordNet has emerged as a useful framework for knowledge discovery and extraction [43–49]. In this research, we adopt Wu and Palmer's similarity measure [50], which has become somewhat of a standard for measuring similarity between words in a lexical ontology. As shown in

(1) Similarity(w1,w2)=2×depth(hw1,w2)deppl(w1,hw1,w2)+deppl(w2,hw1,w2)+2×depth(hw1,w2),

where depth(*h*_*w*_1_,*w*_2__) is the depth of the lowest common hypernym (*h*_*w*_1_,*w*_2__) in a lexical taxonomy, dep_pl_(*w*_1_, *h*_*w*_1_,*w*_2__) and dep_pl_(*w*_2_, *h*_*w*_1_,*w*_2__) denote the number of hops from *h*_*w*_1_,*w*_2__ to *w*_1_ and *w*_2_, respectively.

### 2.2. The Link Grammar

*Link grammar* (LG) [51], designed by Davy Temperley, John Lafferty, and Daniel Sleator, is a syntactic parser of English which builds relations between pairs of words. Given a sentence, LG produces a corresponding syntactic structure, which consists of a set of labeled links connecting pairs of words. The latest version of LG also produces a “constituent representation” (Penn tree-bank style phrase tree) of a sentence (noun phrases, verb phrases, etc.). The parser uses a dictionary of more than 6,000 word forms and has coverage of a wide variety of syntactic constructions. LG is now being maintained under the auspices of the Abiword project [52]. The basic idea of LG is thinking of words as blocks with connectors which form the relations, or called links. These links are used not only to identify the part-of-speech of words but also to describe functions of those words in a sentence in detail. LG can explain the modification relations between different parts of speech and treats a sentence as a sequence of words and consists of a set of labeled links connecting pairs of words. All of the words in the LG dictionary have been defined to describe the way they are used in sentences, and such a system is termed a “lexical system.”

A lexical system can easily construct a large grammar structure, as changing the definition of a word only affects the grammar of the sentence that the word is in. Additionally, expressing the grammar of irregular verbs is simple as the system individually defines each one. As to the grammar of different phrase structures, links that are smooth and conform to semantic structure can be established for every word by using link grammar words to analyze the grammar of a sentence.

All produced links among words obey three basic rules [51].

1. Planarity: the links do not cross to each other.
2. Connectivity: the links suffice to connect all the words of the sequence together.
3. Satisfaction: the links satisfy the linking requirements of each word in the sequence.

**Figure 1:**
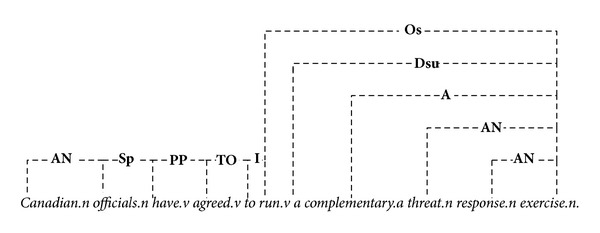
Linkage structures produced by link grammar.

In the sentence “*Canadian officials have agreed to run a complementary threat response exercise*.”, for example, there are** AN** links connect noun-modifiers “*official*” to noun “*Canadian,*” “*exercise*” to “*response,*” and “*exercise*” to “*threat*” as shown in Figure 1. The main words are marked with “*.n*”, “*.v*”, “*.a*” to indicate nouns, verbs, and adjectives. The** A** link connects prenoun (attributive) adjectives to nouns. The link** D** connects determiners to nouns. There are many words that can act as either determiners or noun-phrases such as “*a*” (labeled as “**Ds**”), “*many*” (“**DmC**”), and “*some*” (“**Dm**”), and each of them is corresponding to the subtype of the linking type** D**. The link** O **connects transitive verbs to direct or indirect objects, in which** Os** is a subtype of** O** that connectors mark nouns as being singular.** PP **connects forms of “have” with past participles (“*have agreed*”),** Sp** is a subtype of** S** that connects plural nouns to plural verb forms (**S** connects subject-nouns to finite verbs), and so on.

This simple example illustrates that the linkages imply a certain degree of semantic correlations in the sentence. LG defines more than 100 links; however, in our design, the semantic similarity is extracted from a specific designed linkage-matrix and is evaluated by the WordNet similarity measure; thus, only the connectors contain nonspecific nouns and verbs are reserved. Others links, such as** AL** (which connects a few determiners to following determiners, such as “*both the*” and “*all the*”) and** EC** (which connects adverbs and comparative adjectives, like “*much more*”), are ignored.

## 3. The Grammatical Semantic Similarity Algorithm

This section shows the proposed grammatical similarity algorithm in detail. This algorithm can be a plug-in of normal English natural language processing systems and expert systems. Our approach obtains similarity from semantic and syntactic information contained in the compared natural language sentences. A natural language sentence is considered as a sequence of links instead of separated words and each of which contains a specific meaning. Unlike existing approaches use fixed term set of vocabulary, cooccurrence terms [1–3], or even word orders [8], the proposed approach directly extracts the latent semantics from the same or similar links.

### 3.1. Linking Types

The proposed algorithm determines the similarity of two natural language sentences from the grammar information and the semantic similarity of words that the links contain.  Table 1 shows the selected links, subtypes of links, and the corresponding descriptions used in our approach. The first column is the selected major linking types of* LG*. The second column shows the selected subtypes of the major linking types. If all subtypes of a specific link were selected, it is denoted by “∗.” The dash line identifies that there is no any subtype been selected or exists. This method is divided into three functions. The first part is the linking type extraction. Algorithm 1 accepts a sentence *S* and a set of selected linking types *η* and returns the set of remained linking types and the corresponding information of each link. This is the preprocessing phase; the elements of the returned set are structures that record the links, subtypes of links, and the nouns or verbs of each link.

After preprocessing, Algorithm 2 computes the semantic similarity score of the input sentences. The algorithm accepts two sentences and a set of selected linking types and returns the semantic similarity score, which is formalized to 0~1. In Algorithm 2, lines 1 and 2 call Algorithm 1 to record the links and information of words of sentences *S*_*A*_ and *S*_*B*_ in the sets *LT*_*A*_ and *LT*_*B*_. If *LT*_*A*_∩*LT*_*B*_ ≠ *∅*, it implies that there exist some common or similar links between *S*_*A*_ and *S*_*B*_, which can be regarded as the phrase correlations between the two sentences. In our design, common main links with similar subtypes will form a matrix, named* Grammar_Matrix* (*GM*). Each* GM* implies certain degree of correlations between phrases; the value of each term in* GM* is calculated by the Wu and Palmer algorithm.  Algorithm 3 depicts the details of the evaluation process. In Algorithm 3,* GM* was composed by the common links. Since the number of subtypes varies from each link, we set the links with less subtypes as the rows and the other as the columns. For each row *i*, the maximal term was reserved and forms a* Grammar_Vector* (*GV*), which represents the maximal semantic inclusion of a specific link between *S*_*A*_ and *S*_*B*_.

**Figure 2:**
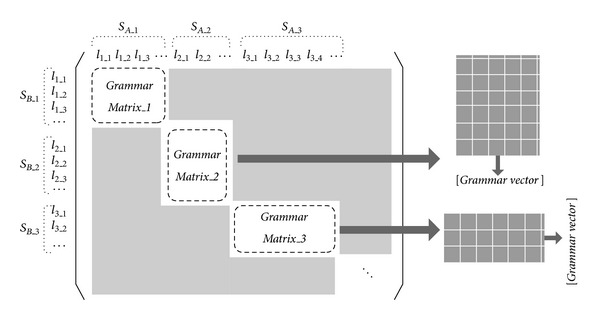
Diagram of grammar matrices and grammar vectors.

**Figure 3:**
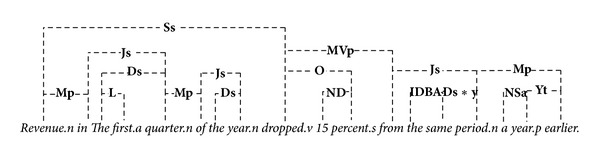
Linkages of sentence* A*.

**Figure 4:**
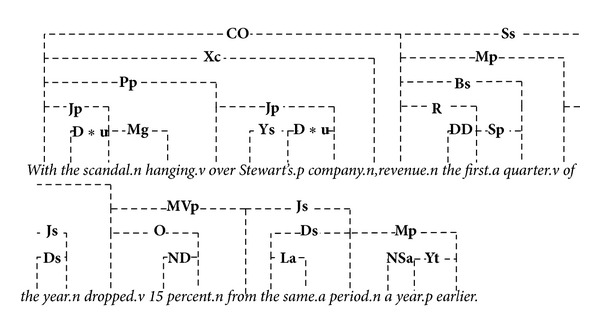
Linkages of sentence* B*.

**Figure 5:**
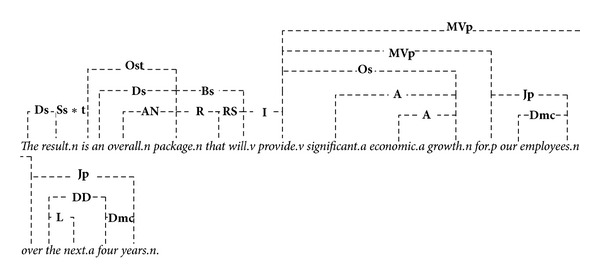
Linkages of sentence* C*.

Figure 2 illustrates the structure of* GMs* and* G* versus *S*_*A*_ and *S*_*B*_ are compared sentences, *S*_*A*_1_ and *S*_*B*_1_ are the first common link and *l*_1_1_, *l*_1_2_, and so forth, are the subtypes of *S*_*A*_1_ and *S*_*B*_1_. Each* GM* represents a correlation of certain phrases since there may exist several similar sublinks in a sentence, in which the corresponding* GV* quantifies the information and extracts latent semantics between these phrases. Algorithm 1 invokes the* LG* function and generates linkages as shown in Figures 3, 4, and 5.

### 3.2. A Work through Example

This section gives an example to demonstrate the proposed similarity algorithm. Let* A* = “*Revenue in the first quarter of the year dropped 15 percent from the same period a year earlier*.”,* B* = “*With the scandal hanging over Stewart's company, revenue the first quarter of the year dropped 15 percent from the same period a year earlier*.”, and* C* = “*The result is an overall package that will provide significant economic growth for our employees over the next four years*.” This example is from the Microsoft Research Paraphrase Corpus (MRPC) [53], which will be introduced in more details in the following section. In this example we compare the semantic similarities between* A-B*,* A-C*, and* B-C*. Algorithm 1 first generates the corresponding linkages for each sentence and the results are shown in Figures 3–5. There are totally 17, 26, and 20 original linkages generated by* LG*. After the preprocessing step, the remaining linkages are (the detailed data structure is omitted here) *LT*_*A*_ = {*Wd*, *S*(*Ss*), *Mp*, *D*(*Ds*), *J*(*Js*)}, *LT*_*B*_ = {*Wd*, *S*(*Ss*), *MP*, *J*(*Jp*, *Js*), *D*(*Ds*, *D*∗*u*)}, and *LT*_*C*_ = {*Wd*, *D*(*Ds*), *DD*, *J*(*Jp*)}, respectively. In Algorithm 2, the compared sentence pair was sent to the* Grammar matrix* (i.e., Algorithm 3) according to their common linking types, and each linking type with their subtypes forms a* Grammar_Matrix*. Tables 2, 3, and 4 show the* GMs* and their word-to-word similarities of pairs* A-B*,* A-C*, and* B-C*. In Table 2, the linking types of *LT*_*A*_∩*LT*_*B*_ are* Wd*,* S*,* Mp*,* D*, and* J*; therefore, there are five* GMs* in pair* A-B*. The first* GM* is a 1 × 1 matrix with row = {*Wd*} and column = {*Wd*}, the second* GM* is also a 1 × 1 matrix with row = {*Ss*} and column = {*Ss*}, the third* GM* is a 3 × 1 matrix with row = {*Mp*} and column = {*Mp*, *Mp*, *Mp*}, the fourth* GM* is a 3 × 4 matrix with row = {*Js*, *Js*, *Js*} and column = {*Jp*, *Jp*, *Js*, *Js*}, and so on. In step 5 of Algorithm 3, we evaluate the single word similarity via the* WordNet* ontology and the* Wu&Palmer* method. The results are also shown in Tables 2–4. This phase evaluates all possible semantics between similar links, and obviously a word may be linked twice or even more in the general case. The next phase reduces each* GM* to a* Grammar_Vector *(*GV*) by reserving the maximal value of each row. Thus in the pair* A-B*, *GV*_*Wd*_ = [1], *GV*_*S*_ = [1], *GV*_*Mp*_ = [1], *GV*_*J*_ = [0.91, 1, 0.91], and *GV*_*D*_ = [0.91, 0.91]. In the pair* A-C*, *GV*_*Wd*_ = [0.31], *GV*_*J*_ = [0.73], *GV*_*Wd*_ = [0.4, 0.55], and *GV*_*Wd*_ = [0.31], *GV*_*J*_ = [0.22, 0.91], and *GV*_*D*_ = [0.71, 0.55] in the pair* B-C*. In the final stage, all elements of GVs are taken the number of the elements' power for balancing the effects of nonevaluated subtypes. The final scores of* A* versus* B* = 0.987,* A* versus* C* = 0.817, and* B* versus* C* = 0.651, respectively.

## 4. Experiments

### 4.1. Experiment with Li's Benchmark

Based on the notion of semantic and syntactic information contributed to the understanding of natural language sentences, Li et al. [8] defined a sentence similarity measure as a linear combination that based on the similarity of semantic vector and word order. A preliminary data set was constructed by Li et al. with human similarity scores provided by 32 volunteers who are all native speakers of English. Li's dataset used 65 word pairs which were originally provided by Rubenstein and Goodenough [60] and were replaced with the definitions from the Collins Cobuild dictionary [61]. The Collins Cobuild dictionary was constructed by a large corpus that contains more than 400 million words. Each pair was rated on the scale of 0.0 to 4.0 according to their similarity of meaning. We used a subset of the 65 pairs to obtain a more even distribution across the similarity range. This subset contains 30 pairs from the original 65 pairs, in which 10 pairs were taken from the range 3~4, 10 pairs from the range 1~3, and 10 pairs from the low level 0~1. We list the full Li's dataset in Table 7.Table 5 shows human similarity scores along with Li et al. [8], an LSA based approach described by O'Shea et al. [54], STS Meth. proposed by Islam and Inkpen [55], SyMSS, a syntax-based measure proposed by Oliva et al. [56], Omiotis proposed by Tsatsaronis et al. [57], and our grammar-based semantic measure. The results indicate that our grammar-based approach achieves a better performance in low and medium similarity sentence pairs (levels 0~1 and 1~3). The average deviation from human judgments in level 0~1 is 0.2, which is better than the most approaches. (Li et al. avg. = 0.356, LSA avg. = 0.496, and SyMSS avg. = 0.266). The average deviation in level 1~3 is 0.208, which is also better than Li et al. and LSA. The result shows that our grammar-based semantic similarity measure achieved a reasonably good performance and the observation is that our approach tries to identify and quantify the potential semantic relation among syntaxes and words, although the common words of the compared sentence pairs are few or even none.

### 4.2. Experiment with Microsoft Research Paraphrase Corpus

In order to further evaluate the performance of the proposed grammar-based approach with a larger dataset, we use the Microsoft Research Paraphrase Corpus [53]. This dataset consists of 5801 pairs of sentences, including 4076 training pairs and 1725 test pairs collected from thousands of news sources on the web over 18 months. Each pair was examined by 2 human judges to determine whether the two sentences in a pair were semantically equivalent paraphrases or not. The interjudge agreement between annotators is approximately 83%. In this experiment, we use different similarity thresholds ranging from 0 to 1 with an interval 0.1 to determine whether a sentence pair is a paraphrase or not. For this task, we computed the proposed approach between the sentences of each pair in the training and test sets and marked as paraphrases only those pairs with similarity value greater than the given threshold. This paper compares the performance of the proposed grammar-based approach against several categories: (1) two baseline methods, a random selection approach that marks each pair as paraphrase randomly, and a traditional VSM-cosine based similarity measure with TF-IDF weighting; (2) corpus-based approaches, the PMI-IR, proposed by Turney at 2001 [62], the LSA [54], STS Meth. [55], SyMSS (with two variations: SyMSS_JCN and SyMSS_Vector) [56], and Omiotis [57]; and (3) lexicon-based approaches, including Jiang and Conrath (JC) at 1997 [63], Leacock et al. (LC) at 1998 [64], Lin (L) at 1998 [65], Resnik (R) [66, 67], Lesk (Lesk) [68], Wu and Palmer (W&P) [50], and Mihalcea et al. (M) at 2006 [69], and (4) machine-learning based approaches, including Wan et al. at 2006 (Wan et al.) [58], Zhang and Patrick at 2005 (Z&P) [70], and Qiu et al. at 2006 (Qiu et al.) [59], which is a SVM [71] based approach.

The results of the evaluation are shown in Table 6. The effectiveness of an information retrieval system is usually measured by two quantities and one combined measure, named “recall” and “precision” rate. In this paper, we evaluate the results in terms of accuracy, and the corresponding precision, recall, and *F*-measure are also shown in Table 6. The performance measures are defined as follows:

(2) $$\begin{array}{c} \text{Precision}=\frac{\text{TP}}{\text{TP}+\text{FP}},\\ \text{Recall}=\frac{\text{TP}}{\text{TP}+\text{FN}},\\ \text{Accuracy}=\frac{\text{TP}+\text{TN}}{\text{TP}+\text{FP}+\text{TN}+\text{FN}},\\ F\text{-Measure}=\frac{2\times \text{Recall}\times \text{Precision}}{\text{Recall}+\text{Precision}}. \end{array}$$

**Figure 6:**
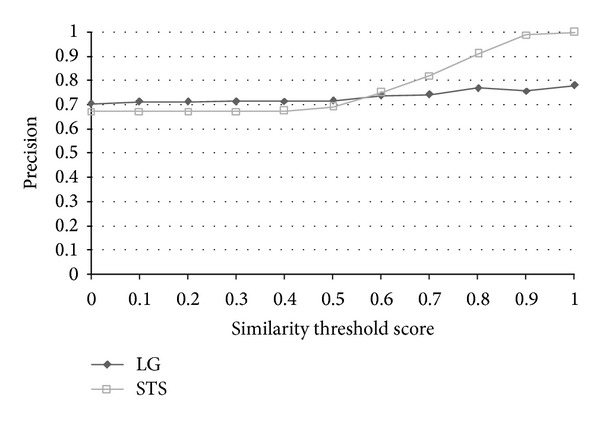
Precision versus similarity threshold curves of STS and LG for eleven different similarity thresholds.

**Figure 7:**
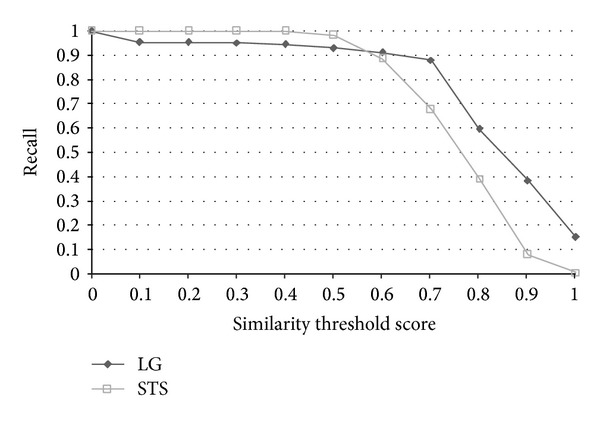
Recall versus similarity threshold curves of STS and LG for eleven different similarity thresholds.

**Figure 8:**
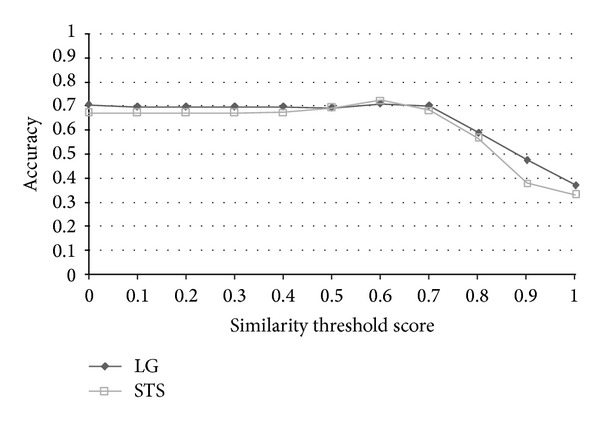
Accuracy versus similarity threshold curves of STS and LG for eleven different similarity thresholds.

**Figure 9:**
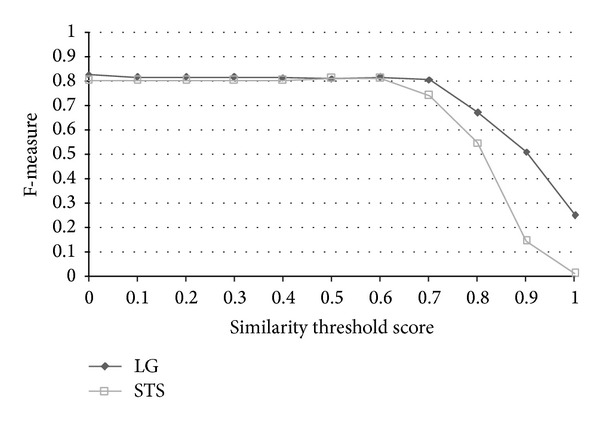
*F*-measure versus similarity threshold curves of STS and LG for eleven different similarity thresholds.

TP, TN, FP, and FN stand for true positive (the number of pairs correctly labeled as paraphrases), true negative (the number of pairs correctly labeled as nonparaphrases), false positive (the number of pairs incorrectly labeled as paraphrases), and false negative (the number of pairs incorrectly labeled as nonparaphrases), respectively. Recall in this experiment is defined as the number of true positives divided by the total number of pairs that actually belong to the positive class, precision is the number of true positives divided by the total number of pairs labeled as belonging to the positive class, accuracy is the number of true results (true positive + true negative) divided by the number of all pairs, and *F*-measure is the geometric mean of recall and precision. After evaluation, the best similarity threshold of accuracy is 0.6. The results indicate that the grammar-based approach surpasses all baselines, lexicon-based, and most of the corpus-based approaches in terms of accuracy and *F*-measure. We must mention that the results of each approach listed above were based on the best accuracy through all thresholds instead of under the same similarity threshold. STS Meth. [55] achieved the best accuracy 72.64 with similarity threshold 0.6, SyMSS_JCN and SyMSS_Vector were two variants of SyMSS [56] who accomplished the best performance in similarity threshold 0.45, and moreover, the best similarity thresholds of Omiotis [57], Mihalcea et al. [69], random selection, and VSM-cosine based similarity measures were 0.2, 0.5, 0.5, and 0.5, respectively. In all lexicon and corpus-based approaches, STS Meth. Reference [55] earns the best similarity score 72.64 and the similarity threshold 0.6 is also reasonable, besides only the STS Meth. Reference [55] has provided detailed recall, precision, accuracy, and *F*-measured values with various thresholds. The following compares our grammar-based approach with STS Meth. [55] in thresholds 0~1.  Figure 6 shows the precision versus similarity threshold curves of STS Meth. and grammar-based method for eleven different similarity thresholds. Figures 7, 8, and 9 depict the recall, accuracy, and *F*-measure versus similarity threshold curves of STS Meth. and grammar-based method, respectively.

As acknowledged by Islam and Inkpen [55] and Corley and Mihalcea [72], semantic similarity measure for short texts/sentences is a necessary step in the paraphrase recognition task, but not always sufficient. In the Microsoft Research Paraphrase Corpus, sentence pairs judged to be nonparaphrases may still overlap significantly in information content and even wording. For example, the Microsoft Research Paraphrase Corpus contains the following sentence pairs.

**Example 1**

(*1)* “*Passed in 1999 but never put into effect, the law would have made it illegal for bar and restaurant patrons to light up*.”

(*2)* “*Passed in 1999 but never put into effect, the smoking law would have prevented bar and restaurant patrons from lighting up, but exempted private clubs from the regulation*.”

**Example 2**

(*1)* “*Though that slower spending made 2003 look better, many of the expenditures actually will occur in 2004*.”

(*2)* “*Though that slower spending made 2003 look better, many of the expenditures will actually occur in 2004, making that year's shortfall worse*.”

Sentences in each pair are highly related to each other with common words and syntaxes, however, they are not considered as paraphrases and are labeled as 0 in the corpus (paraphrases are labeled as 1). For this reason, we believe that the numbers of false positive (FP) and true negative (TN) are not entirely correct and may affect the correctness of precision, *F*-measure but accuracy and recall. The result shows that the proposed grammar-based approach outperforms the result by Islam and Inkpen [55] with thresholds 0.6~1.0 (0.91 versus 0.89 and 0.88 versus 0.68 of recall with thresholds 0.6 and 0.7; 0.71 versus 0.72, 0.70 versus 0.68, and 0.59 versus 0.57 of accuracy in thresholds 0.6, 0.7, and 0.8, resp.), which is a reasonable range in determining whether a sentence pair is a paraphrase or not.

## 5. Conclusions

This paper presents a grammar and semantic corpus based similarity algorithm for natural language sentences. Traditional IR technologies may not always determine the perfect matching without obvious relation or concept overlap between two natural language sentences. Some approaches deal with this problem via determining the order of words and the evaluation of semantic vectors; however, they were hard to be applied to compare the sentences with complex syntax as well as long sentences and sentences with arbitrary patterns and grammars. The proposed approach takes advantage of corpus-based ontology and grammatical rules to overcome this problem. The contributions of this work can be summarized as follows: (1) to the best of our knowledge, the proposed algorithm is the first measure of semantic similarity between sentences that integrates the word-to-word evaluation to grammatical rules, (2) the specific designed* Grammar_Matrix* will quantify the correlations between phrases instead of considering common words or word order, and (3) the use of semantic trees offered by WordNet increases the chances of finding a semantic relation between any nouns and verbs, and (4) the results demonstrate that the proposed method performed very well both in the sentences similarity and the task of paraphrase recognition. Our approach achieves a good average deviation for 30 sentence pairs and outperforms the results obtained by Li et al. [8] and LSA [54]. For the paraphrase recognition task, our grammar-based method surpasses most of the existing approaches and limits the best performance in a reasonable range of thresholds.

## Figures and Tables

**Algorithm 1:**
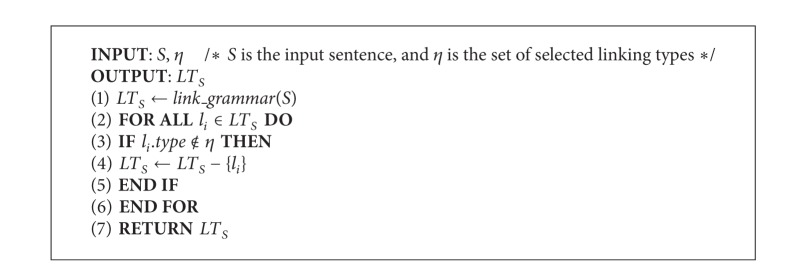
*Linking types*.

**Algorithm 2:**
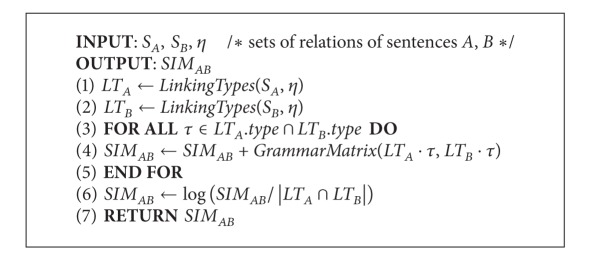
*Semantic sentence similarity*.

**Algorithm 3:**
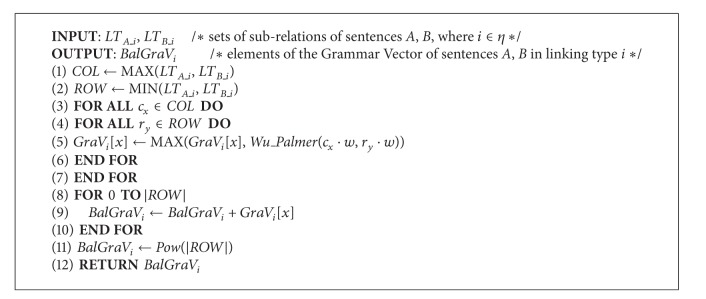
*Grammarmatrix*.

**Table 1:** Selected linkages used in the algorithm; superscript *ξ* denotes the optional linking types.

Links	Subtypes	Descriptions
*A*	—	*A* connects pre-noun adjectives to nouns, such as “*delicious food*” and “*black dog*”.

*AN*	—	*AN* connects noun-modifiers (singular noun) to nouns, such as “*bacon toast*” and “*seafood pasta*”.

*B*	*Bs* *Bp*	*B* connects noun to a verb in restrictive relative clauses, and *Bs* and *Bp* are used to enforce noun-verb agreement in subject-type relative clauses (relative clauses without “,”), such as “*He will see his son who lives in New York*”.
	*Bs* *d*	*Bs* *d* is used for words “∗*ever*” like “*whatever*” and “*whoever*”.
	*Bs*∗*w*	*Bs*∗*w* is used for object-type questions with words like “*which*", “*what*", “*who,*" and so forth.

*D*	*Ds*	*D* connects determiners to nouns, *Ds* connects singular determiners like “*a*”, “*one*” to nouns, such as “*a cat*”, “*one month.*”
	*Dm* *c*	*Dm* *c* connects plural determiners like “*some*”, “*many*” to countable nouns.
	*D*∗*u*	*D*∗*u* connects mass determiners to uncountable nouns.

*DD* ^*ξ*^	—	*DD* is used to connect definite determiners like “*the*”, “*his*”, “*my*” to number expressions and adjectives acting as nouns, such as “*my two sisters*”.

*DG* ^*ξ*^	—	*DG* is used to connect “*the*" with proper nouns.

*DT* ^*ξ*^	*DT* *i*	*DT* is used to connect determiners with nouns in certain idiomatic time expressions, such as “*last week*" and “*this Tuesday*". *DTi* connects time expressions like “*next*”, “*last*” to nouns.
	*DT* *n*	*DT* *n* connects time expressions like “*this*”, “*every*” to nouns, such as “*every Sunday*”.

*GN*	—	*GN* connects expressions where proper nouns are introduced by a common noun, such as “*the famous physicist Edward Witten*”.

*J*	∗	*J* connects prepositions to their objects. Proper nouns, common nouns, accusative pronouns, and words that can act as noun-phrases have “*J*” link.

*JG* ^*ξ*^	—	*JG* connects prepositions like "*of*" and "*for*" to proper-nouns, such as “*the WIN7 of Microsoft*”.

*M*	*Mp*	*M* connects nouns to postnominal modifiers such as prepositional phrases, participle modifiers, prepositional relatives, and possessive relatives, in which *Mp* works in prepositional phrases modifying nouns.

*MG*	—	*MG* allows certain prepositions to modify proper nouns, such as the above sentence.

*MX* ^*ξ*^	∗	*MX* links nouns to postnominal noun modifiers surrounded by commas, such as “*the teacher*, *who*…”

*O* ^*ξ*^	∗	*O* connects transitive verbs to nouns, pronouns, and words that can act as noun-phrases or heads of noun-phrases, such as “*told him*”, “*saw him*”.

*R*	—	*R* is used to connect nouns to relative clauses, such as “*The man who*…”.

*S* ^*ξ*^	*Ss*	*S* connects subject nouns to finite verbs. The subtype *Ss* connects singular nouns words to singular verb, such as “*She sings very well*”.
	*Sp*	*Sp* connects plural nouns to plural verb forms, such as “*The monkeys ate these apples.*”
	*Ss*∗*w*	*Ss*∗*w* is used for question words that act as noun-phrases in subject-type questions, such as “*Who is there.*”

*SI* ^*ξ*^	∗	*SI* is used in subject-verb inversion, such as “*Which one do you want.*”

*Us* ^*ξ*^	—	*Us* is used with nouns that both satisfy the determiner requirement and subject-object requirement, such as “*We check that per hour.*”

*WN*	—	*WN* connects “*when*" phrases back to time-nouns, such as “*This month when I was in Taipei*…”

*YP* ^*ξ*^	—	*YP* connects plural noun forms ending in “*s*” to “*'*”, such as “*The students' parents.*”

**Table 2:** The *GMs* of sentence *A* versus sentence *B*.

*A*/*B*	*S*_*B*_		*Wd*	*S*	*Mp*	*J*				*D*		
*S*_*A*_	Subtypes and words		*Wd*	*Ss*	*Mp*	*Jp*	*Jp*	*Js*	*Js*	*D*∗*u*	*D*∗*u*	*Ds*
			revenue	revenue- dropped	revenue- of	with- scandal	over- company	of- year	from- period	the- scandal	‘s- company	the- period

*Wd*	*Wd*	revenue	1	—	—	—	—	—	—	—	—	—

*S*	*Ss*	revenue- dropped	—	1	—	—	—	—	—	—	—	—

*Mp*	*Mp*	revenue-in	—	—	1	—	—	—	—	—	—	—
	*Mp*	quarter-of	—	—	0.33	—	—	—	—	—	—	—
	*Mp*	period-earlier	—	—	0.33	—	—	—	—	—	—	—

*J*	*Js*	in-quarter	—	—	—	0.33	0.67	0.83	0.91	—	—	—
	*Js*	from-period	—	—	—	0.33	0.36	0.91	1	—	—	—
	*Js*	of-year	—	—	—	0.31	0.77	1	0.91	—	—	—

*D*	*Ds*	the-quarter	—	—	—	—	—	—	—	0.33	0.67	0.91
	*Ds*	the-year	—	—	—	—	—	—	—	0.31	0.77	0.91

**Table 3:** The *GMs* of sentence *A* versus sentence *C*.

*A*/*C*	*S*_*C*_		*Wd*	*J*		*D*	
*S*_*A*_	Subtypes and words		*Wd*	*Jp*	*Jp*	*Ds*	*Ds*
			result	for-employees	over-years	the-result	an-package

*Wd*	*Wd*	revenue	0.31	—	—	—	—

*J*	*Js*	in-quarter	—	0.73	0.83	—	—
	*Js*	from-period	—	0.18	0.91	—	—
	*Js*	of-year	—	0.22	0.9	—	—

*D*	*Ds*	the-quarter	—	—	—	0.4	0.4
	*Ds*	the-year	—	—	—	0.33	0.55

**Table 4:** The *GMs* of sentence *B *versus sentence *C*.

*C*/*B*	*S*_*B*_		*Wd*	*J*				*D*			
*S*_*C*_	Subtypes and words		*Wd*	*Jp*	*Jp*	*Js*	*Js*	*D*∗*u*	*D*∗*u*	*Ds*	*Ds*
			revenue	with- scandal	over- company	of- year	from- period	the- scandal	‘s- company	the- period	the- year

*Wd*	*Wd*	result	0.31	—	—	—	—	—	—	—	—

*J*	*Jp*	for-employees	—	0	0.22	0.22	0.11	—	—	—	—
	*Jp*	over-years	—	0.31	0.33	0.9	0.91	—	—	—	—

*D*	*Ds*	the-result	—	—	—	—	—	0.71	0.33	0.5	0.33
	*Ds*	an-package	—	—	—	—	—	0.33	0.55	0.4	0.55

**Table 5:** Benchmark number and the results compared with Li et al. [8], LSA [54], STS Meth. [55], SyMSS [56], Omiotis [57], and our grammar-based approach.

R&G number	Human	Li_McLean	LSA	STS Meth.	SyMSS	Omiotis	LG
1	0.01	0.33	0.51	0.06	0.32	0.11	0.22
5	0.01	0.29	0.53	0.11	0.28	0.10	0.06
9	0.01	0.21	0.51	0.07	0.27	0.10	0.35
13	0.10	0.53	0.53	0.16	0.27	0.30	0.32
17	0.13	0.36	0.58	0.26	0.42	0.30	0.41
21	0.04	0.51	0.53	0.16	0.37	0.24	0.44
25	0.07	0.55	0.60	0.33	0.53	0.30	0.07
29	0.01	0.34	0.51	0.12	0.31	0.11	0.20
33	0.15	0.59	0.81	0.29	0.43	0.49	0.07
37	0.13	0.44	0.58	0.20	0.23	0.11	0.07
41	0.28	0.43	0.58	0.09	0.38	0.11	0.02
47	0.35	0.72	0.72	0.30	0.24	0.22	0.25
48	0.36	0.64	0.62	0.34	0.42	0.53	0.79
49	0.29	0.74	0.54	0.15	0.39	0.57	0.38
50	0.47	0.69	0.68	0.49	0.35	0.55	0.07
51	0.14	0.65	0.73	0.28	0.31	0.52	0.39
52	0.49	0.49	0.70	0.32	0.54	0.60	0.84
53	0.48	0.39	0.83	0.44	0.52	0.5	0.18
54	0.36	0.52	0.61	0.41	0.33	0.43	0.32
55	0.41	0.55	0.70	0.19	0.33	0.43	0.38
56	0.59	0.76	0.78	0.47	0.43	0.93	0.62
57	0.63	0.7	0.75	0.26	0.50	0.61	0.82
58	0.59	0.75	0.83	0.51	0.64	0.74	0.94
59	0.86	1	1	0.94	1	1	1
60	0.58	0.66	0.83	0.6	0.63	0.93	0.89
61	0.52	0.66	0.63	0.29	0.39	0.35	0.08
62	0.77	0.73	0.74	0.51	0.75	0.73	0.94
63	0.59	0.64	0.87	0.52	0.78	0.79	0.95
64	0.96	1	1	0.93	1	0.93	1

**Table 6:** Results of the grammar-based and competitive methods on the Microsoft Research Paraphrase Corpus.

Category	Metric	Accuracy	Precision	Recall	*F*-measure
Corpus-based	PMI-IR	69.90	70.20	95.20	81.00
	LSA	68.40	69.70	95.20	80.50
	STS Meth.	72.64	74.65	89.13	81.25
	SyMSS_JCN	70.87	74.70	84.17	79.02
	SyMSS_Vector	70.82	74.15	90.32	81.44
	Omiotis	69.97	70.78	93.40	80.52

Lexicon-based	JC	69.30	72.20	87.10	79.00
	LC	69.50	72.40	87.00	79.00
	Lesk	69.30	72.40	86.60	78.90
	L	69.30	71.60	88.70	79.20
	W&P	69.00	70.20	92.10	80.00
	R	69.00	69.00	96.40	80.40
	M	70.30	69.60	97.70	81.30

Machine learning-based	Wan et al. [58]	75.00	77.00	90.00	83.00
	Z&P	71.90	74.30	88.20	80.70
	Qiu et al. [59]	72.00	72.50	93.40	81.60

Baselines	Random	51.30	68.30	50.00	57.80
	VSM	65.40	71.60	79.50	75.30

	LG	71.02	73.90	91.07	81.59

**Table 7:** Li's Dataset [8].

Number	Word pair	Raw sentences	Human Sim.
1	cord : smile	(1) Cord is strong, thick string. (2) A smile is the expression that you have on your face when you are pleased or amused, or when you are being friendly.	0.0100

2	rooster : voyage	(1) A rooster is an adult male chicken. (2) A voyage is a long journey on a ship or in a spacecraft.	0.0050

3	noon : string	(1) Noon is 12 o'clock in the middle of the day. (2) String is thin rope made of twisted threads, used for tying things together or tying up parcels.	0.0125

4	fruit : furnace	(1) Fruit or a fruit is something which grows on a tree or bush and which contains seeds or a stone covered by a substance that you can eat. (2) A furnace is a container or enclosed space in which a very hot fire is made, for example to melt metal, burn rubbish, or produce steam.	0.0475

5	autograph : shore	(1) An autograph is the signature of someone famous which is specially written for a fan to keep. (2) The shores or shore of a sea, lake, or wide river is the land along the edge of it.	0.0050

6	automobile : wizard	(1) An automobile is a car. (2) In legends and fairy stories, a wizard is a man who has magic powers.	0.0200

7	mound : stove	(1) A mound of something is a large rounded pile of it. (2) A stove is a piece of equipment which provides heat, either for cooking or for heating a room.	0.0050

8	grin : implement	(1) A grin is a broad smile. (2) An implement is a tool or other pieces of equipment.	0.0050

9	asylum : fruit	(1) An asylum is a psychiatric hospital. (2) Fruit or a fruit is something which grows on a tree or bush and which contains seeds or a stone covered by a substance that you can eat.	0.0050

10	asylum : monk	(1) An asylum is a psychiatric hospital. (2) A monk is a member of a male religious community that is usually separated from the outside world.	0.0375

11	graveyard : madhouse	(1) A graveyard is an area of land, sometimes near a church, where dead people are buried. (2) If you describe a place or situation as a madhouse,you mean that it is full of confusion and noise.	0.0225

12	glass : magician	(1) Glass is a hard transparent substance that is used to make things such as windows and bottles. (2) A magician is a person who entertains people by doing magic tricks.	0.0075

13	boy : rooster	(1) A boy is a child who will grow up to be a man. (2) A rooster is an adult male chicken.	0.1075

14	cushion : jewel	(1) A cushion is a fabric case filled with soft material, which you put on a seat to make it more comfortable. (2) A jewel is a precious stone used to decorate valuable things that you wear, such as rings or necklaces.	0.0525

15	monk : slave	(1) A monk is a member of a male religious community that is usually separated from the outside world. (2) A slave is someone who is the property of another person and has to work for that person.	0.0450

16	asylum : cemetery	(1) An asylum is a psychiatric hospital. (2) A cemetery is a place where dead people's bodies or their ashes are buried.	0.0375

17	coast : forest	(1) The coast is an area of land that is next to the sea. (2) A forest is a large area where trees grow close together.	0.0475

18	grin : lad	(1) A grin is a broad smile. (2) A lad is a young man or boy.	0.0125

19	shore : woodland	(1) The shores or shore of a sea, lake, or wide river is the land along the edge of it. (2) Woodland is land with a lot of trees.	0.0825

20	monk : oracle	(1) A monk is a member of a male religious community that is usually separated from the outside world. (2) In ancient times, an oracle was a priest or priestess who made statements about future events or about the truth.	0.1125

21	boy : sage	(1) A boy is a child who will grow up to be a man. (2) A sage is a person who is regarded as being very wise.	0.0425

22	automobile : cushion	(1) An automobile is a car. (2) A cushion is a fabric case filled with soft material, which you put on a seat to make it more comfortable.	0.0200

23	mound : shore	(1) A mound of something is a large rounded pile of it. (2) The shores or shore of a sea, lake, or wide river is the land along the edge of it.	0.0350

24	lad : wizard	(1) A lad is a young man or boy. (2) In legends and fairy stories, a wizard is a man who has magic powers.	0.0325

25	forest : graveyard	(1) A forest is a large area where trees grow close together. (2) A graveyard is an area of land, sometimes near a church, where dead people are buried.	0.0650

26	food : rooster	(1) Food is what people and animals eat. (2) A rooster is an adult male chicken.	0.0550

27	cemetery : woodland	(1) A cemetery is a place where dead people's bodies or their ashes are buried. (2) Woodland is land with a lot of trees.	0.0375

28	shore : voyage	(1) The shores or shore of a sea, lake, or wide river is the land along the edge of it. (2) A voyage is a long journey on a ship or in a spacecraft.	0.0200

29	bird : woodland	(1) A bird is a creature with feathers and wings, females lay eggs, and most birds can fly. (2) Woodland is land with a lot of trees.	0.0125

30	coast : hill	(1) The coast is an area of land that is next to the sea. (2) A hill is an area of land that is higher than the land that surrounds it.	0.1000

31	furnace : implement	(1) A furnace is a container or enclosed space in which a very hot fire is made, for example to melt metal, burn rubbish or produce steam. (2) An implement is a tool or other piece of equipment.	0.0500

32	crane : rooster	(1) A crane is a large machine that moves heavy things by lifting them in the air. (2) A rooster is an adult male chicken.	0.0200

33	hill : woodland	(1) A hill is an area of land that is higher than the land that surrounds it. (2) Woodland is land with a lot of trees.	0.1450

34	car : journey	(1) A car is a motor vehicle with room for a small number of passengers. (2) When you make a journey, you travel from one place to another.	0.0725

35	cemetery : mound	(1) A cemetery is a place where dead people's bodies or their ashes are buried. (2) A mound of something is a large rounded pile of it.	0.0575

36	glass : jewel	(1) Glass is a hard transparent substance that is used to make things such as windows and bottles. (2) A jewel is a precious stone used to decorate valuable things that you wear, such as rings or necklaces.	0.1075

37	magician : oracle	(1) A magician is a person who entertains people by doing magic tricks. (2) In ancient times, an oracle was a priest or priestess who made statements about future events or about the truth.	0.1300

38	crane : implement	(1) A crane is a large machine that moves heavy things by lifting them in the air. (2) An implement is a tool or other piece of equipment.	0.1850

39	brother : lad	(1) Your brother is a boy or a man who has the same parents as you. (2) A lad is a young man or boy.	0.1275

40	sage : wizard	(1) A sage is a person who is regarded as being very wise. (2) In legends and fairy stories, a wizard is a man who has magic powers.	0.1525

41	oracle : sage	(1) In ancient times, an oracle was a priest or priestess who made statements about future events or about the truth. (2) A sage is a person who is regarded as being very wise.	0.2825

42	bird : crane	(1) A bird is a creature with feathers and wings, females lay eggs, and most birds can fly. (2) A crane is a large machine that moves heavy things by lifting them in the air.	0.0350

43	bird : cock	(1) A bird is a creature with feathers and wings, females lay eggs, and most birds can fly. (2) A cock is an adult male chicken.	0.1625

44	food : fruit	(1) Food is what people and animals eat. (2) Fruit or a fruit is something which grows on a tree or bush and which contains seeds or a stone covered by a substance that you can eat.	0.2425

45	brother : monk	(1) Your brother is a boy or a man who has the same parents as you. (2) A monk is a member of a male religious community that is usually separated from the outside world.	0.0450

46	asylum : madhouse	(1) An asylum is a psychiatric hospital. (2) If you describe a place or situation as a madhouse, you mean that it is full of confusion and noise.	0.2150

47	furnace : stove	(1) A furnace is a container or enclosed space in which a very hot fire is made, for example, to melt metal, burn rubbish, or produce steam. (2) A stove is a piece of equipment which provides heat, either for cooking or for heating a room.	0.3475

48	magician : wizard	(1) A magician is a person who entertains people by doing magic tricks. (2) In legends and fairy stories, a wizard is a man who has magic powers.	0.3550

49	hill : mound	(1) A hill is an area of land that is higher than the land that surrounds it. (2) A mound of something is a large rounded pile of it.	0.2925

50	cord : string	(1) Cord is strong, thick string. (2) String is thin rope made of twisted threads, used for tying things together or tying up parcels.	0.4700

51	glass : tumbler	(1) Glass is a hard transparent substance that is used to make things such as windows and bottles. (2) A tumbler is a drinking glass with straight sides.	0.1375

52	grin : smile	(1) A grin is a broad smile. (2) A smile is the expression that you have on your face when you are pleased or amused, or when you are being friendly.	0.4850

53	serf : slave	(1) In former times, serfs were a class of people who had to work on a particular person's land and could not leave without that person's permission. (2) A slave is someone who is the property of another person and has to work for that person.	0.4825

54	journey : voyage	(1) When you make a journey, you travel from one place to another. (2) A voyage is a long journey on a ship or in a spacecraft.	0.3600

55	autograph : signature	(1) An autograph is the signature of someone famous which is specially written for a fan to keep. (2) Your signature is your name, written in your own characteristic way, often at the end of a document to indicate that you wrote the document or that you agree with what it says.	0.4050

56	coast : shore	(1) The coast is an area of land that is next to the sea. (2) The shores or shore of a sea, lake, or wide river is the land along the edge of it.	0.5875

57	forest : woodland	(1) A forest is a large area where trees grow close together. (2) Woodland is land with a lot of trees.	0.6275

58	implement : tool	(1) An implement is a tool or other pieces of equipment. (2) A tool is any instrument or simple piece of equipment that you hold in your hands and use to do a particular kind of work.	0.5900

59	cock : rooster	(1) A cock is an adult male chicken. (2) A rooster is an adult male chicken.	0.8625

60	boy : lad	(1) A boy is a child who will grow up to be a man. (2) A lad is a young man or boy.	0.5800

61	cushion : pillow	(1) A cushion is a fabric case filled with soft material, which you put on a seat to make it more comfortable. (2) A pillow is a rectangular cushion which you rest your head on when you are in bed.	0.5225

62	cemetery : graveyard	(1) A cemetery is a place where dead people's bodies or their ashes are buried. (2) A graveyard is an area of land, sometimes near a church, where dead people are buried.	0.7725

63	automobile : car	(1) An automobile is a car. (2) A car is a motor vehicle with room for a small number of passengers.	0.5575

64	midday : noon	(1) Midday is 12 o'clock in the middle of the day. (2) Noon is 12 o'clock in the middle of the day.	0.9550

65	gem : jewel	(1) A gem is a jewel or stone that is used in jewellery. (2) A jewel is a precious stone used to decorate valuable things that you wear, such as rings or necklaces.	0.6525
